# A Symptomatic Mucinous Cystic Neoplasm of the Mesentery: A Case Report

**DOI:** 10.7759/cureus.59660

**Published:** 2024-05-05

**Authors:** Maya Amy, Sanford E Roberts, Maria F Arisi, Robin Collingwood, Lillias Maguire

**Affiliations:** 1 Surgery, Drexel University College of Medicine, Philadelphia, USA; 2 Surgery, University of Pennsylvania, Philadelphia, USA; 3 Pathology and Laboratory Medicine, University of Pennsylvania, Philadelphia, USA

**Keywords:** sigmoid tumor, colorectal neoplasia, mesenteric tumor, surgical case reports, mucinous cystic neoplasm

## Abstract

Mucinous cystic neoplasms (MCNs) are rare tumors primarily observed in the pancreas but occasionally found in other locations such as the retroperitoneum, ovary, liver, and spleen. These neoplasms are histologically classified based on the degree of dysplasia, with some associated with invasive carcinoma. Colorectal surgeons infrequently encounter MCNs. Mesenteric MCNs pose a diagnostic challenge secondary to their atypical location, subtle histology, and lack of specific biochemical markers. In this context, we present a case involving a 68-year-old female who initially presented with an assumed ovarian mass. Subsequent exploration revealed a 12 cm MCN situated in the sigmoid mesentery, a location seldom associated with these tumors. The patient underwent laparotomy with successful resection and recovery. Histopathological analysis confirmed the neoplasm's mucinous epithelium with a complex papillary architecture. Immunohistochemical staining supported the diagnosis, revealing positivity for CK7, SATB2, and CDX2.

## Introduction

Mucinous cystic neoplasms (MCN) are rare tumors classically arising in the pancreas. Less frequently, they have been identified in the retroperitoneum, ovary, liver, and spleen [1]. MCNs are classified histologically into those having low-grade dysplasia, high-grade dysplasia, or those associated with invasive carcinoma [1]. These lesions are rarely seen by colorectal surgeons and due to their unusual nature, case reports provide an opportunity for advancement of knowledge. We present a 68-year-old female with an enlarging presumed ovarian mass found to have an MCN of the sigmoid mesentery.

## Case presentation

A 68-year-old G2P1 female presented with a complex medical history, including hyperlipidemia, hypertension, osteoporosis, partial idiopathic seizures managed with Keppra, and pre-diabetes. She had a longstanding history of an adnexal cystic mass initially noted in 2013 on an MRI pelvis with contrast at an outside hospital. At that time the mass was read as a likely paraovarian cyst or lymphocele. It is unclear if any follow-up was recommended at that time but the patient recalls being told that the results were “benign.”

She continued in her usual state of health until 2020 when she presented to her primary care physician for bilateral hip pain. A pelvic MRI (musculoskeletal protocol) was performed, which demonstrated a pelvic mass concerning ovarian neoplasm. She was referred to OB-GYN at that time. A pelvic transvaginal ultrasound (US) was ordered to further characterize the mass. The US demonstrated an enlarged left ovary containing a complex cystic mass measuring 7.5x5.8x6.7 cm. Given the stability in size from the prior imaging, it was thought to be less likely malignant; however, tumor marker CA-125 was sent, which was within normal range. She was also ordered to complete a colonoscopy at this time; however, this was delayed due to various patient factors. 

She followed up with an OB-GYN in December 2021 who recommended a laparoscopic left salpingo-oophorectomy for the purpose of diagnosis versus ongoing surveillance with pelvic MRI and CA-125 lab work. The patient elected to pursue surveillance. She remained symptomatic from the mass at this time. Her surveillance care was referred to a gynecologic oncologist. A repeat MRI was performed in January of 2021, which demonstrated a 6.1x7.2x6.4 cm hypointense lesion within the left adnexa. It contained fluid/debris level as well as small cystic foci along its wall. Additionally, there was a small 0.5x0.9x1.0 cm enhancing mural nodule along the posterior cyst wall. The overall impression was that the lesion may represent a serous cystadenoma; however, the presence of the small enhancing mural nodule raised the possibility of a serous borderline tumor. 

In August of 2022, she began experiencing dysuria, urinalysis demonstrated hematuria, and she was referred to urology who recommended a CT urogram, which demonstrated possible enlargement of the posterior nodule of the mass but no focal findings to explain the hematuria. Cystoscopy was recommended. 

She followed up with her gynecologic oncologist in September 2022 who recommended a robotic total hysterectomy and bilateral salpingo-oophorectomy. Hysterectomy was recommended at that time given the concern that her fibroid uterus may have been contributing to some of her urinary symptoms. Shortly after her visit, the patient decided to cancel her surgery. 

She completed her cystoscopy, which revealed normal urethra and bladder with no tumors, foreign bodies, or stones. Her colonoscopy demonstrated a 10 mm polyp at the recto-sigmoid junction, which was resected; otherwise, there were no abnormal findings. 

She completed a surveillance pelvic MRI in March 2023, which demonstrated an interval increase in the size of the left adnexal mass to 8.7x8.6 cm. At this time, it was noted that the structure appeared adjacent to but separate from the left ovary. Given these findings, she represented to the gynecologic oncologist clinic and rescheduled her surgery. She was taken to the operating room (OR) in April 2023. In the OR upon inspection, it was clear that the mass was separate from her GYN anatomy and, in fact, arising from the sigmoid colon. Surgical oncology was consulted intra-operatively. Given that the patient had not received bowel preparation or consented to a bowel resection, the decision was made to conclude the operation and refer her to a colorectal surgeon. In May of 2023, she saw a colorectal surgeon who performed staging CT chest/abdomen and pelvis that showed the known mass as well as ordered a repeat colonoscopy. 

**Figure 1:**
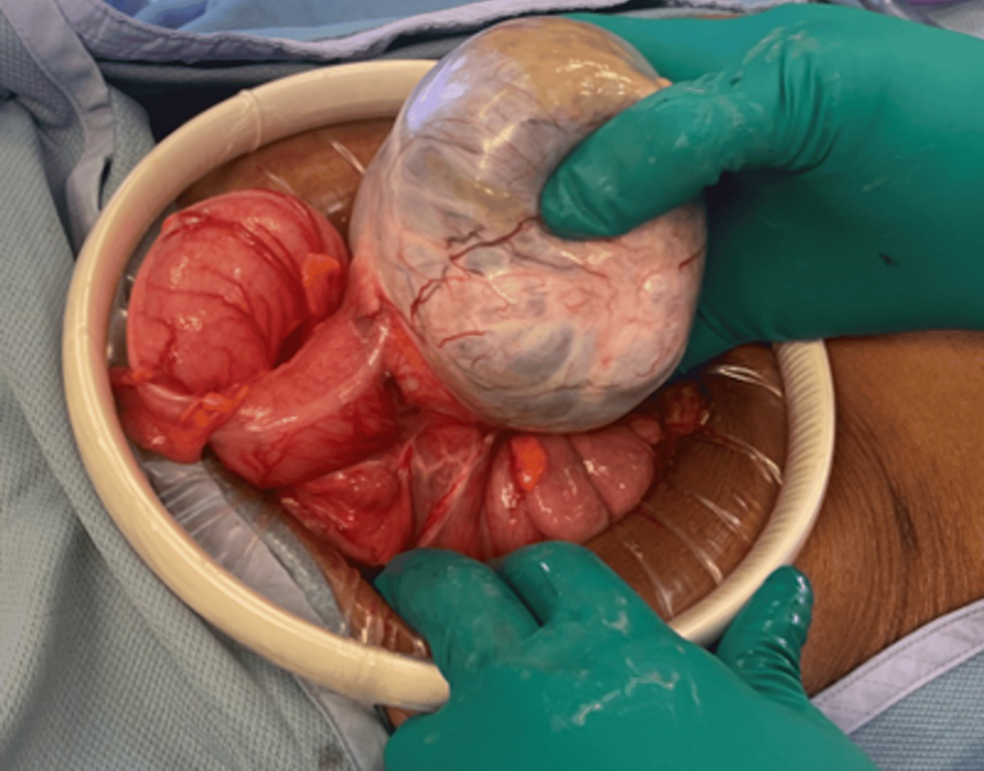
Intra-operative photograph of sigmoid colon mass

Repeat colonoscopy demonstrated an extramural non-obstructing large mass in the sigmoid colon. The mass was non-circumferential. A date for surgery was set; she was instructed to perform a bowel preparation and was subsequently taken to the OR in September 2023 for an exploratory laparotomy. In the OR, a lower midline laparotomy was created. There was a large obvious mobile mass in the left pelvis arising from the sigmoid colon (Figure 1). The colon was transected several centimeters proximal and distal to the mass and the mesentery was divided using an electrocautery device. A side-to-side stapled colocolostomy was performed, a final inspection was performed, and the abdomen was closed. The patient recovered well post-operatively and was discharged on post-operative day two.

**Figure 2:**
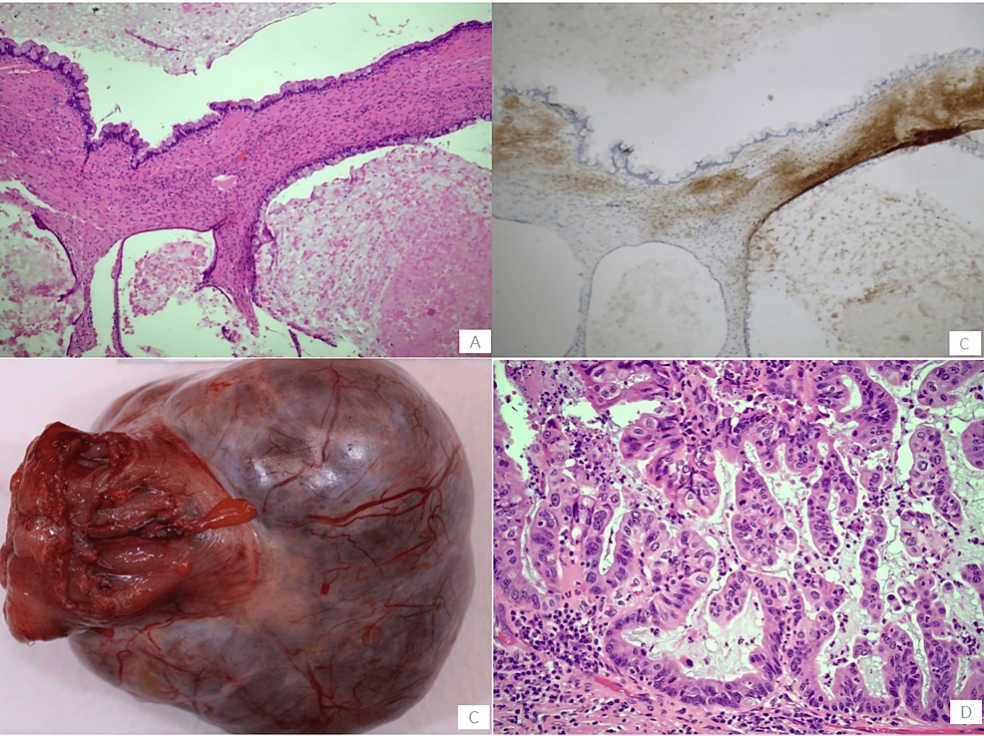
Gross and histopathology of MCN A. Low-grade mucinous epithelium with ovarian stroma (H&E, 10x). B. Ovarian stroma (ER IHC, 10x). C. Gross lesion. D. Areas of high-grade dysplasia (H&E, 20x). MCN, mucinous cystic neoplasm

The patient’s surgical pathology revealed a 12 cm cyst lined by mucinous epithelium with intestinal and foveolar subtypes with a complex papillary architecture and extensive cytologic atypia. Immunohistochemical stains with adequate controls were performed (Figure 2). The lesional cells were positive for CK7, SATB2 (focally), and CDX2 (focally) and negative for CK20 and PAX-8. Immunohistochemical staining for the estrogen receptor (ER) highlighted ovarian-type stroma. There was no involvement of the adherent colon. The final diagnosis was an MCN of the mesentery.

## Discussion

MCNs are rare lesions occurring almost exclusively in the pancreas but may manifest at extra-pancreatic sites, including the mesentery, retroperitoneum, ovary, liver, and spleen [1]. They have been rarely described in the mesentery [2]. The epidemiology of MCNs highlights a predominance in adult females [1,3-5]. Histologically, MCNs are similar to ovarian mucinous neoplasms and are characterized by a mucin-secreting flat, columnar, or cuboidal epithelium associated with an underlying ovarian-like stroma [2,4]. Identification of the ovarian-like stroma can be achieved by positive immunohistochemical staining for vimentin, α-smooth muscle actin, and desmin or via identification of spindle-shaped cells with myofibroblastic proliferation [2,4].

Various theories exist regarding the origin of MCNs. Their pathogenesis has been linked to ectopic ovarian tissue, mucinous metaplasia of epithelial cells, or origins from teratomas [1-6]. The most widely accepted theory suggests that they arise from peritoneal invaginations, leading to the formation of inclusion cysts, followed by mucinous metaplasia of epithelial cells [2-4]. 

Mesenteric MCNs pose a diagnostic challenge secondary to the anatomic location, often subtle histologic findings, lack of specific immunohistochemical markers, and rarity. As in this case, it may be difficult to pinpoint the site of origin of these tumors on imaging. Aspiration cytology and needle biopsy are not recommended as the cystic nature of MCNs can result in non-diagnostic results and may be associated with recurrence [6]. Furthermore, the intra-operative frozen section does not appear to have any utility, as the final diagnosis requires specialized staining for accurate post-operative final pathology [4]. 

Mesenteric MCNs are exceedingly rare, with reported cases exhibiting a significant malignant potential [5]. Given this malignant potential, as well as their high recurrence rate and risk of hemorrhage or infection, complete excision is the recommended treatment [4,6]. Post-operative histopathology remains essential for accurate diagnosis and grading [4,6]. To date, there is no clear data on whether it is beneficial to do a more extensive lymph node harvest at the time of surgery. Both open and laparoscopic approaches have been described as safe options for resection [4].

## Conclusions

In conclusion, this case report highlights the diagnostic and therapeutic challenges encountered in the treatment of MCNs, particularly when they present in rare locations. These tumors classically arise in the pancreas or liver; an occurrence in the mesentery is exceptionally uncommon and presents a significant challenge in achieving an accurate preoperative diagnosis. Our patient’s presumed diagnosis of an ovarian cyst highlights this difficulty. Histopathological and immunohistochemical analyses are instrumental in confirming the diagnosis as MCNs can mimic other neoplastic processes. Given the potential for malignancy and high recurrence rate, complete excision is crucial in the treatment of MCNs arising in the mesentery.
